# Signal Pathways and Markers Involved in Acute Lung Injury Induced by Acute Pancreatitis

**DOI:** 10.1155/2021/9947047

**Published:** 2021-08-28

**Authors:** Jialin Zhou, Pengcheng Zhou, Yingyi Zhang, Guangzhi Wang, Zhe Fan

**Affiliations:** ^1^Department of General Surgery, The Third People's Hospital of Dalian, Dalian Medical University, Dalian, China; ^2^School of Medicine, Southeast University, Nanjing, China; ^3^Department of General Surgery, The Second Hospital of Dalian Medical University, Dalian, China

## Abstract

Acute pancreatitis (AP) is a common acute abdominal disease with a mortality rate of about 30%. Acute lung injury (ALI) is a common systemic complication of acute pancreatitis, with progressive hypoxemia and respiratory distress as the main manifestations, which can develop into acute respiratory distress syndrome or even multiple organ dysfunction syndrome (MODS) in severe cases, endangering human health. In the model of AP, pathophysiological process of the lung can be summarized as oxidative stress injury, inflammatory factor infiltration, and alveolar cell apoptosis. However, the intrinsic mechanisms underlying AP and how it leads to ALI are not fully understood. In this paper, we summarize recent articles related to AP leading to ALI, including the signal transduction pathways and biomarkers of AP-ALI. There are factors or pathway aggravating ALI, the JAK2-STAT3 signaling pathway, NLRP3/NF-*κ*B pathway, mitogen-activated protein kinase, PKC pathway, neutrophil protease (NP)-LAMC2-neutrophil pathway, and the P2X7 pathway, and there are important transcription factors in the NRF2 signal transduction pathway which could give researchers better understanding of the underlying mechanisms controlling AP and ALI and lay the foundation for finally curing ALI induced by AP.

## 1. Introduction

Acute pancreatitis (AP) is an acute inflammatory process of the pancreas, which can injure not only local peripancreatic tissue but remote organs and systems as well [1]. The acute inflammatory state of the pancreas usually follows an infection, which may lead to multisystem organ dysfunction, including acute lung injury (ALI) [2–4]. During this pathophysiological process, cytokines and inflammatory mediators are released in large quantities, activating multiple signaling pathways which cause damage to the body. However, the underlying mechanism is not completely clear. In recent years, the signaling pathways mediating the occurrence of severe AP (SAP) have become better known, and it has now been shown that multiple signaling pathways are involved in the biological processes of alveolar endothelial cell proliferation, differentiation, and apoptosis caused by AP. In this paper, we summarize the roles of seven pathways and related biomarkers in AP-ALI which have increased our understanding of the development of the disease and provided novel therapeutic approaches for its treatment.

## 2. JAK2-STAT3 Signaling Pathway

The Janus kinase/signal transducer and activator of transcription (JAK/STAT) pathway has previously been shown to play a role in tumorigenesis. Interleukin-6 (IL-6) is a proinflammatory cytokine that preferentially activates STAT3 and has a role in both initiating and exacerbating the inflammatory process. During inflammation, adhesion molecules, substances expressed on endothelial cells (ECs), contribute to the recruitment and migration of leukocytes to the subendothelial stroma [5].

AP can induce the expression of intercellular adhesion molecule-1 (ICAM-1) through the JAK2/STAT3 signaling pathway, and the induction of ICAM-1 is associated with leukocyte adhesion and migration, leading to amplification of endothelial cell injury and inflammatory response. In addition, AP can activate IL-6 and tumor necrosis factor (TNF)-*α*, further activating the JAK2/STAT3 pathway, leading to ICAM-1 activation and promoting the upregulation of nuclear factor-*κ*B (NF-*κ*B), which in turn induces the development of ALI [6].

**Figure 1:**
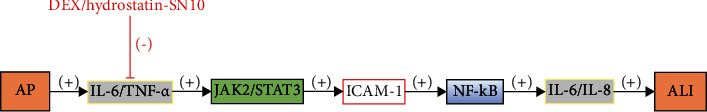
AP can activate IL-6 and TNF-*α*, further activating the JAK2/STAT3 pathway, leading to ICAM-1 activation and promoting the upregulation of NF-*κ*B, which in turn induces the development of ALI. DEX/hydrostatin-SN10 inhibits this pathway.

It has been suggested that high levels of tumor necrosis factor alpha (TNF-*α*) and nuclear factor-*κ*B (NF-*κ*B) may induce the expression of ICAM-1 and thus be involved in the development of SAP-ALI [6]. Dexamethasone treatment attenuates SAP-induced upregulation of TNF-*α* and NF-*κ*B [7]. Dexamethasone treatment may reduce cytokine production by inhibiting ICAM-1, which may be a cause of its anti-inflammatory effect [6]. IL-6 inhibits proliferation, promotes apoptosis, and contributes to lung injury by activating the JAK2/STAT3 signaling pathway [8–10]. A short mutant peptide of hydrostatin-SN10 (peptide sequence, DEQHLETELH) extracted from snake venom inhibits AP-ALI by inhibiting IL-6 induced by JAK2/STAT3 signaling (Figure 1).

## 3. NLRP3/NF-*κ*B Pathway

Protein 3 (NLRP3) inflammasome is a substance containing NACHT, LRR, and PYD domains that leads to the production of IL-1*β* and IL-18 by sensing pathogen and danger-related molecular patterns (PAMPs and DAMPs) [11]. NF-*κ*B signaling is an important initial step in initiating NLRP3 activation, and reactive oxygen species (ROS) generated by NF-*κ*B-mediated inflammation are also a risk signal for NLRP3 activation [12]. NLRP3 activation is followed by ASC recruitment, activation of cysteine protease-1 (caspase-1), and induction of pro-IL-1*β* or pro-IL-18 processing and maturation [13, 14]. Thus, both signals, NLRP3 and NF-*κ*B, act together to induce the activation of cytokines (e.g., IL-1*β*) that promote ALI.

Lack of functional Toll-like receptor 4 (TLR4) leads to a decreased NF-*κ*B response and reduced production of proinflammatory mediators, ameliorating lung inflammation in mice and alveolar macrophages [15]. In addition, monocyte chemotactic protein-1 (MCP-1) is an important factor that has been shown to induce AP as a direct target of NF-*κ*B [16, 17].

**Figure 2:**
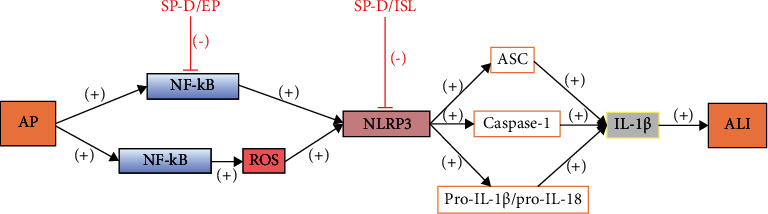
AP may cause NF-*κ*B signaling initiation and also NF-*κ*B-mediated activation of ROS produced by inflammation, which leads to activation of NLRP3 and consequent recruitment of ASC, activation of caspase-1, and induction of pro-IL-1*β* or pro-IL-18 into mature forms, which can then induce the activation of cytokines (e.g., IL-1*β*) and thereby promote lung injury in ALI. ISL inhibits NLRP3 activation, and EP inhibits NF-*κ*B activation, both of which attenuate severe pancreatitis-associated ALI.

Surfactant protein D (SP-D) inhibits SAP-induced ALI and pancreatic injury. It may do so through a pathway that inhibits the activation of NLRP3, inflammasome, and NF-*κ*B signaling [18]. Isoflavonopoietin (ISL), a flavonoid derived from licorice, can inhibit NLRP3 pathway by activating Nrf2, inhibiting NF-*κ*B, and also inhibiting NLRP3 activation [19, 20]. Ethylpyruvate inhibits NF-*κ*B activation and downregulates downstream inflammatory cytokine expression in SAP rats and attenuates severe pancreatitis-associated ALI [21] (Figure 2).

## 4. Mitogen-Activated Protein Kinase (MAPK)

Mitogen-activated protein kinase (MAPK), including P38MAPK, c-Jun N-terminal kinase (JNK), and extracellular signal-regulated kinase (ERK), is a member of the serine/threonine kinase family and plays an important role in inflammation, tumorigenesis, cell proliferation, apoptosis, differentiation, and stress responses [22–25]. ERK is activated in response to ischemic injury, such as hemorrhagic shock and stroke, and its activation may lead to cell damage and death [26]. The p38MAPK is an important signal transduction enzyme that regulates gene transcription and translation by transducing extracellular signals into cells and is primarily involved in the release of inflammatory cytokines/mediators in the pathogenesis of inflammatory diseases such as ALI and AP [25, 27]. AP-activated TNF-*α* induces ALI via p-JNK/MAPK and p-ERK/MAPK in the lung, while p38MAPK can be activated by a variety of extracellular stimuli, such as inflammatory mediators, heat injury, and ultraviolet light. p38MAPK is activated, and chemokines are increased after hemorrhagic shock and can contribute to the development of ALI [28].

**Figure 3:**
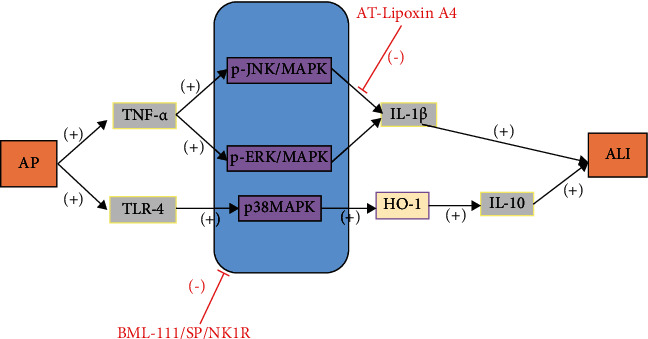
AP-activated TNF-*α* induces ALI via p-JNK/MAPK and p-ERK/MAPK. AP-activated TLR-4 induces ALI via p38MAPK-induced upregulation of HO-1. AT-Lipoxin A4 inhibits the p-JNK/MAPK and p-ERK/MAPK pathways, BML-111 blocks phosphorylation of JNK, ERK, and p38MAPK, and SP/NK1R may prevent ALI by regulating LTB4 production.

MAPK activation can cause multiorgan dysfunction after hemorrhagic shock (MODS) [29, 30]. Therefore, prevention and control of the MAPK signaling pathway may be an important way to prevent hemorrhagic shock-induced ALI and multiorgan dysfunction. BML-111 blocks phosphorylation of JNK, ERK, and p38MAPK in hemorrhagic shock [31]. AT-Lipoxin A4 inhibits the p-JNK/MAPK and p-ERK/MAPK pathways [32]. Substance P (SP)/neurokinin-1 receptor (NK1R) may regulate pancreatitis leukotriene B4 (LTB4) production via the MAPK signaling pathway, and LTB4 may regulate neutrophil reverse transendothelial migration (rTEM) in AP, which further promotes AP-ALI [32, 33]. Upregulation of microRNA-542-5p downregulates the expression of P21-associated kinase 1 (PAK1), and downregulation of PAK1 may contribute to inhibition of the MAPK signaling pathway [33]. Lipoprotein A4 (LXA4) blocks ALI by inhibiting the inflammatory pathways of NF-*κ*B and p38MAPK and by upregulating cytoprotective heme oxygenase-1 (HO-1) [34] (Figure 3).

## 5. PKC Pathway

Protein kinase C (PKC) is a member of the family of phospholipid-dependent serine/threonine kinases. It consists of at least several isoforms [35, 36]. Conventional PKC alleles (*α*, *β*I, *β*II, and *β*), novel PKC isoforms (*δ*, *ε*, *η*, and *θ*), and other PKC isoforms (*λ* subclass, *γ* subclass), as well as four PKC isoforms (*α*, *δ*, *ε*, and *ζ*), each with a unique activation pattern, have been identified in pancreatic follicular cells [37]. Experimental studies have shown that inflammatory mediators are overproduced and released in the lung through a PKC-dependent pathway [38]. The PKC pathway is an important signaling pathway that can be activated by inflammatory cytokines. src-inhibited C kinase substrate (SSeCKS), a PKC substrate and a major inflammatory response protein that is significantly overexpressed in ALI, selectively binds to signaling proteins such as PKC to disrupt endothelial cell permeability [39]. The PKC pathway regulates cytoskeletal protein activity and endothelial cell barrier function by modulating its downstream substrate SSeCKS. PKC-mediated upregulation of SSeCKS activates F-actin, which leads to NF-*κ*B activation in HPMEC, resulting in ALI [40]. Because rescue of aquaporin 5 (AQP-5) and matrix metalloproteinase 9 (MMP-9) and inhibition of apoptosis may lead to NF-*κ*B attenuation [41], we speculate that NF-*κ*B may be a key mediator of apoptosis, AQP-5/MMP-9, and PKC/SSeCKS/F-actin signaling pathways during AP-induced ALI.

**Figure 4:**
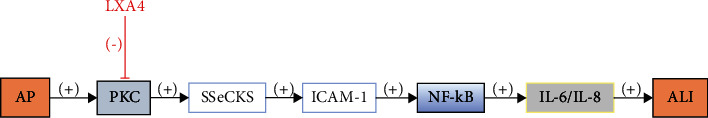
AP can cause PKC-mediated upregulation of SSeCKS leading to activation of F-actin and promote upregulation of NF-*κ*B, which in turn induces the development of ALI. LXA4 can inhibit this pathway.

SP regulates LTB4 production via the PKC*α*/MAPK pathway, which in turn promotes AP-ALI via neutrophil rTEM [42]. LXA4 effectively promotes F-actin remodeling and regulates its expression in pulmonary microvascular endothelial cells both in vivo and in vitro by inhibiting the PKC/SSeCKS signaling pathway [43] (Figure 4).

## 6. NPs-LAMC2-Neutrophil Pathway

**Figure 5:**
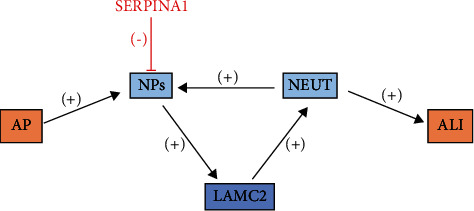
Enzymatic cleavage of NPs releases LAMC2 fragments that in turn promote neutrophil recruitment and induce acute phase NP production. LAMC2 has been reported to be overexpressed and associated with ALI in its early stages. It can negatively regulate the activity of NPs to attenuate AP-induced ALI.

Laminin gamma 2 (LAMC2) and Serpin Family A Member 1 (SERPINA1) are associated with collagen-containing extracellular matrix, leukocyte-cell adhesion, and regulation of endopeptidases [44]. It has been suggested that the LAMC2 fragment is released by the cleavage of NP enzymes; importantly, the released LAMC2 fragment in turn promotes neutrophil recruitment [45]. This would induce the production of NP in the acute phase. LAMC2 has been reported to be overexpressed and associated with the early stages of ALI. Thus, NPs, LAMC2, and neutrophils may form positive-feedback loops in the pathogenesis of SAP-ALI. Upregulation of LAMC2 expression in SAP-ALI lung tissue may be due to increased expression of LAMC2 in SAP-ALI lung tissue. SERPINA1 is a serine protease inhibitor that negatively regulates the activity of NPs. The high expression level of serine protease inhibitor B1 (serpinB1) in SAP-ALI lung tissue and its possible association with the aggregation of high numbers of neutrophils and monocytes in the lung suggest that it may be a novel biomarker of disease severity. Emodin may exert a protective effect by negatively regulating NP activity and blocking NPs-LAMC2 in SAP-ALI. Neutrophil-altered loops significantly attenuate AP-induced ALI [46] (Figure 5).

## 7. P2X7 Pathway

**Figure 6:**
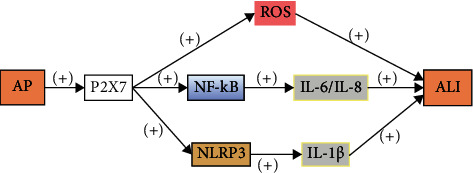
AP promotes the activation of P2X7, which stimulates multiple signaling pathways, including ROS, NLRP3, and NF-*κ*B, the latter of which produces large amounts of inflammatory mediators that induce the development of ALI.

SAP is a sterile inflammatory condition characterized by the release of large amounts of proinflammatory cytokines from damaged glandular follicle cells [47]. The purinergic receptor P2X7 is a member of the P2X family of ATP-gated cation channels and an important molecule involved in the inflammatory response [48]. Activation of P2X7 stimulates multiple signaling pathways such as reactive oxygen species (ROS), MAPKs, and NF-*κ*B, which produce large amounts of inflammatory mediators [49, 50]. Recent studies have shown that P2X7 can effectively stimulate inflammatory activation of NLRP3 [51–53]. Numerous studies have shown that P2X7R is mainly expressed in rodent pancreatic ductal cells and regulates calcium signaling and ion transport [54–56]. Cabili et al. found evidence that NLRP3 receptors are also expressed in the exocrine glands of animals. Alveoli in the pancreas exhibit low functionality and a marked lack of P2X7 receptors for purinergic receptor signaling, but pancreatic duct cells express high amounts of various P2 receptors, especially P2X7 receptors [57]. In addition, SAP is usually initially aseptic, which predisposes to necrosis of the glandular follicle cells [58]. A sterile inflammatory response mediated by damage-associated molecular patterns (DAMP) released from necrotic glandular follicle cells predisposes animals to pancreatic injury, which acts through plasma membrane P2X7 receptors [59]. In addition, the P2X7/NLRP3 pathway is activated 12 h after pancreatic injury. However, inflammation is largely time-course dependent, suggesting that induction of P2X7 is associated with the severity of pancreatitis (Figure 6).

## 8. NRF2 Signal Transduction Pathway

The nuclear factor erythroid-2-related factor 2 (Nrf2) pathway is thought to be a survival pathway for the mitigation of oxidative damage. Nrf2 is a protective antioxidant that regulates cellular oxidation and reduction homeostasis, and for oxidative stress, the Nrf2 pathway can be modulated to treat SAP [60–62]. Activation of Nrf2 is an important strategy to inhibit ROS generation and control oxidative stress. Furthermore, Nrf2 is an important regulator in ALI [63–65]. Under basal conditions, Nrf2 is present in the cytoplasm as a component of the cell and binds to Kelch-like ECH-associated protein 1 (Keap1), which is ultimately degraded. However, when organisms are under oxidative stress, Nrf2 dissociates from Keap1, a process that can be achieved through various mechanisms such as oxidative modification of cysteine thiols in classical Keap1 and phosphorylation of specific amino acid residues of Nrf2 through multiple protein kinase pathways [66].

**Figure 7:**
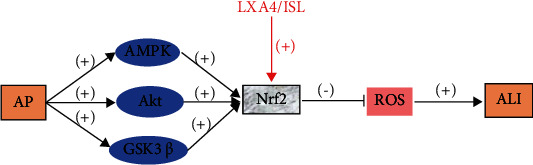
AP activates AMPK, which activates Akt kinase and GSK3*β* to mediate Nrf2 activation. TNF-*α* activates the Nrf2 signaling pathway and HO-1, and activation of Nrf2 may inhibit ROS production and control oxidative stress, which in turn inhibits ALI. LXA4/ISLT can treat ALI/ARDS by activating Nrf2.

The intracellular energy sensor AMP-activated protein kinase (AMPK) is a kinase which is considered to be upstream of Nrf2 and is of interest because of the relationship with redox homeostasis and energy metabolism [67]. In addition, another mechanism of AMPK-mediated Nrf2 activation may include Akt kinase and glycogen synthase kinase 3 beta (GSK3*β*) [68]. TNF-*α* can activate the Nrf2 signaling pathway and its downstream gene HO-1; furthermore, LXA4 in HPMEC, as a potent anti-inflammatory and novel antioxidant mediator, can further promote Nrf2 expression. LXA4 may attenuate AP-induced inflammation and ROS by regulating the Nrf2 pathway. When injected intraperitoneally, isoliquiritigenin, with a chalcone structure (4,20,40-trihydroxy chalcone), can treat ALI/Acute Respiratory Distress Syndrome (ARDS) associated with gram-negative bacterial infections by activating Nrf2 [69] (Figure 7).

## 9. Summary and Outlook

AP leads to the continuous activation of various signaling pathways in ALI, as shown in recent studies; ALI was assessed as shown in the indices listed in Table 1. By inhibiting the transduction of the aggravated AP-ALI pathway and promoting the transduction of the attenuated AP-ALI pathway, the secretion of proinflammatory factors can be reduced, pulmonary edema can be reduced, and certain therapeutic effects can be achieved. The discovery of precise and effective target inhibitors still depends on the study of genes and proteins involved in the pathway, but the study of diagnostic genes and proteomics of inflammatory diseases is still at a preliminary stage. Therefore, ALI induced by more surgical critical care conditions needs to be more thoroughly explored, especially in ischemia/reperfusion [70, 71], sepsis [72], trauma [73], and transfusion [74, 75]. In the future, we plan to analyze the interaction between various proteins and genes to deepen our understanding of the mechanism of inflammatory diseases and provide for effective diagnosis and treatments.

## Figures and Tables

**Table 1:** Indices of ALI induced by acute pancreatitis.

Model	Models induced	Indices of ALI
Mouse	Cerulein [4, 43] L-arginine [33]	Lung tissue *W*/*D* ratio [4, 43] MPO activity [33] BALF and cell analysis [4] MDA assays [33] Histological analysis [4, 33, 43]
Rat	Deoxycholic acid sodium salt [6] Sodium taurocholate [21, 31, 46]	Lung tissue *W*/*D* ratio [21] MPO activity [21] MDA assays [21] Histological analysis [21, 31, 46]

MPO: myeloperoxidase; MDA: malondialdehyde; *W*/*D* ratio: wet/dry weight ratio; BALF: bronchoalveolar lavage fluid.

## Data Availability

The data used to support the findings of this study are available from the corresponding author upon request.
